# Circulating inflammatory cytokines in relation to the risk of renal cell carcinoma: A gender‐specific two‐sample Mendelian randomization study

**DOI:** 10.1002/cam4.6658

**Published:** 2023-10-30

**Authors:** Shuixiang Tao, Yiwei Lin, Shengqiang Huang, Shen Lin, Ke Jin, Hong Chen

**Affiliations:** ^1^ Department of Urology Shaoxing People's Hospital (Zhejiang University Shaoxing Hospital) Shaoxing Zhejiang China; ^2^ Department of Urology, the First Affiliated Hospital Zhejiang University School of Medicine Hangzhou Zhejiang China; ^3^ Department of Urology The People's Hospital of Pujiang County Jinhua Zhejiang China

**Keywords:** cytokines, Eotaxin, Mendelian randomization, renal cell carcinoma

## Abstract

**Background:**

Currently there is no specific molecular biomarker for the diagnosis and treatment of renal cell carcinoma (RCC). Here we performed a gender‐specific two‐sample Mendelian randomization analysis to systematically assess the effects of circulating cytokines on RCC.

**Methods:**

We have employed cis‐quantitative trait loci as instrumental variables for the protein levels and expression of circulating cytokines. We estimated the causal effects of circulating cytokines on RCC risk in males and females with several Mendelian randomization methods.

**Results:**

We observed a significant causal effect of Eotaxin on the increased risk of RCC in males (Odds ratio [OR] = 2.546, 95% confidence interval [CI] = 1.617–4.010, *p* value = 5.496 × 10–5), but not in females (OR = 1.352, 95% CI = 0.766–2.388, *p* value = 0.298). Besides, we also identified several cytokines as potentially associated with RCC in males including RANTES, MCP3, PDGFbb, TRAIL, and several other cytokines as potentially associated with RCC in females including sICAM and SCGFb.

**Conclusion:**

Our study highlighted that a higher level of circulating Eotaxin is causally associated with an increased risk of RCC in males but not in females. Further studies are needed to elucidate the exact mechanism and its potential application in the prognosis and treatment of RCC.

## 1 INTRODUCTION

Renal cell carcinoma (RCC) is the most common malignant neoplasm of the kidney with an incidence rate of over 4%.^1^, ^2^, ^3^ RCC accounts for around 2%–3% of all cancers and is estimated to have an overall mortality rate of 30%–40%.^4^, ^5^ At diagnosis of RCC, 20%–30% of patients present with metastatic disease.^2^ Around half of all RCC cases were diagnosed during abdominal ultrasound or CT scanning for other purposes due to the late onset of symptoms.^6^ Therefore, it appears as an urgent need to define the factors involved in the development and progression of RCC for early diagnosis and treatment.

Accumulated evidence suggested inflammation as a hallmark of cancer.^7^ Chronic inflammation has been reported to be associated with several types of cancer.^8^, ^9^ Specific inflammatory cytokines were also found to have an effect on cancer development in previous observational studies.^10^ However, the exact role of cytokines in RCC is still unclear. Currently, there are no specific molecular biomarkers for renal cell carcinoma early detection or the targeted treatment.^11^ Screening for inflammatory cytokines that play an important role in RCC may help to improve the diagnosis and treatment of RCC.

Inflammatory cytokines have been much discussed as potential targets for cancer treatment; however, most of the evidence was from observational studies. Studies with higher level evidence are needed to support their causal roles in cancer development. However, a randomized control trial (RCT) is not optional because of feasibility and ethical considerations.

Mendelian randomization (MR) is an epidemiological method that employs single nucleotide polymorphisms (SNPs) as instrumental variables to proxy certain exposures.^12^ Individuals from a certain population can be divided into different subgroups using their genetic variants associated with the exposures of interest. Thus, we can compare the genetically defined subgroups that are different from the others in these exposures. As the SNPs were assigned randomly during conception, which means they are independent of the environment or other factors, the MR‐based study mimics an RCT design.^13^ Estimates from MR were less subject to reverse causality or confounding factors, which were common limitations related to observational studies.

In this study, we have performed two‐sample MR analyses assessing the causal effects of circulating inflammatory cytokines on the risk of RCC in males and females. We have identified that the gene expression and protein level of circulating Eotaxin are causally associated with RCC in males, but not in females.

## 2 METHODS

### 2.1 Study design

**FIGURE 1:**
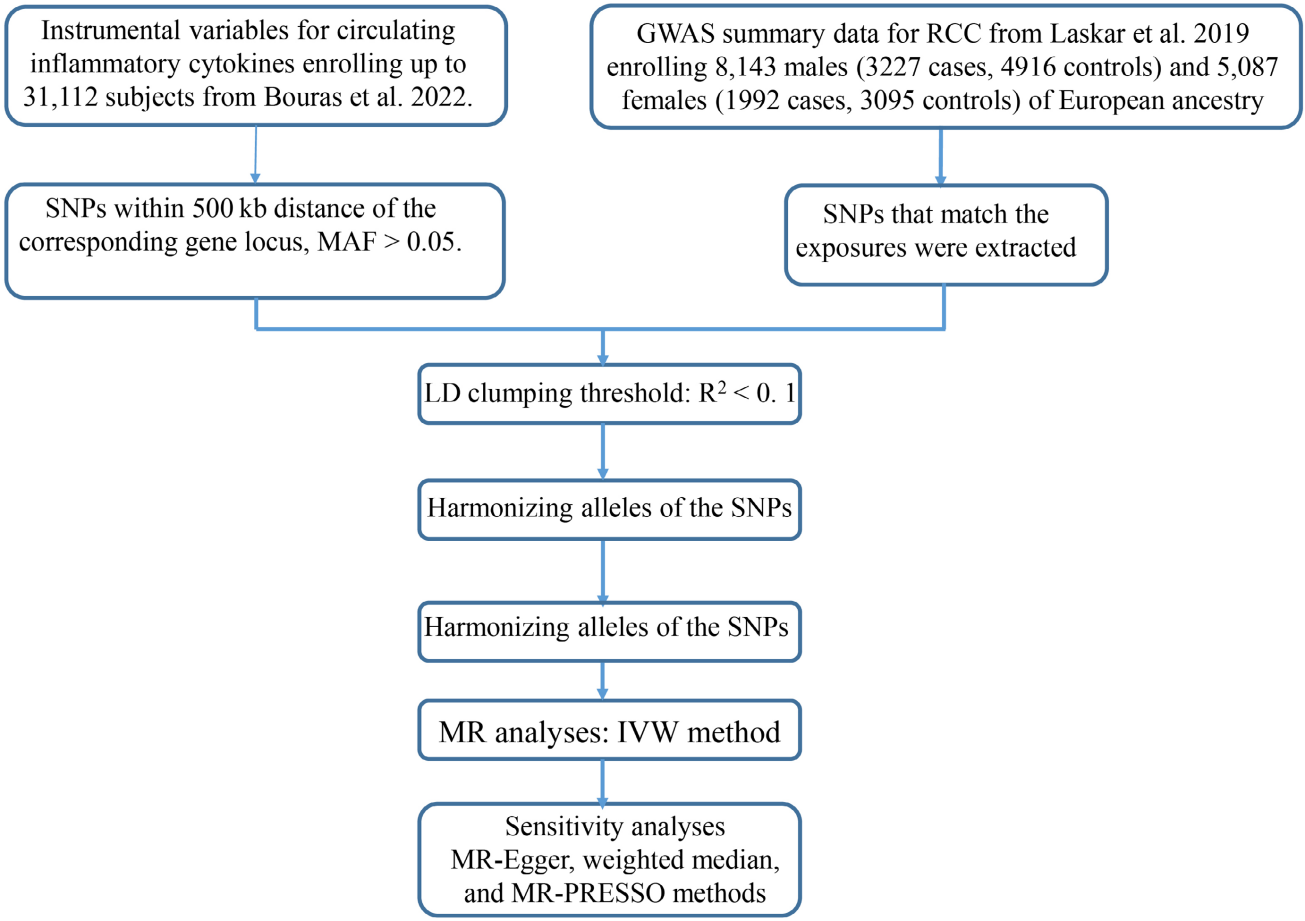
Diagrams showing the study design overview. MR, Mendelian randomization; SNP, single nucleotide polymorphism; GWAS, genome‐wide association study; RCC, renal cell carcinoma; LD, linkage disequilibrium; MAF, minor allele frequency; IVW, inverse‐variance weighted.

An overview of the study design can be found in Figure 1.

### 2.2 Instrumental variable selection

The instrumental variables associated with the circulating cytokines were obtained from a previous publication.^14^ In brief, the genetic association of inflammatory cytokines were from three publicly available genome‐wide association studies (GWASs) datasets: one with samples of up to 13,365 individuals from the Northern Finland Birth Cohort 1966 (NFBC1966), the Cardiovascular Risk in Young Finns (YFS) study and FINRISK 1997 and 2002^15^, ^16^; one from a GWAS of up to 21,758 individuals from the SCALLOP consortium; and another one from a GWAS of up to 3301 individuals from the INTERVAL study.^17^, ^18^ Associations of SNPs with inflammatory cytokines from the three sources were pooled with a meta‐analysis.^14^ To minimize the risk of horizontal pleiotropy, the cis‐protein quantitative trait locus (cis‐pQTL) and cis‐expression quantitative trait locus (cis‐eQTL) were used for the MR analyses. The cis‐pQTLs and cis‐eQTLs were defined as SNPs within 500 kb distance of the corresponding gene locus, the cis‐pQTLs are significantly associated with circulating cytokine levels at *p* < 1 × 10^−4^, and the cis‐eQTLs are significantly associated with the gene's expression across all tissues at *p* < 1 × 10^−4^, and with the circulating cytokine concentrations at *p* < 0.05.^14^ SNPs with a minor allele frequency of less than 0.05 were excluded from the analyses. SNPs in linkage disequilibrium (LD) were identified and excluded with LD clumping by using a threshold of *R* ^2^ < 0.1. Furthermore, SNPs that are associated with other cytokines (*p* < 5 × 10^−8^) were also excluded from the MR analyses.

### 2.3 Outcome sources

The associations of the SNPs on RCC were obtained from summary statistics of a GWAS meta‐analysis from the International Agency for Research on Cancer (IARC).^19^ The sex‐specific GWAS meta‐analysis included 8143 males (3227 cases and 4916 controls) and 5087 females (1992 cases and 3095 controls) of European ancestry from 11 studies from 18 countries.^19^, ^20^ The samples were genotyped with HumanHap 317 k, 550 or 610Q, Omni5, and OmniExpress arrays.^19^ Thirty cytokines had enough cis‐pQTLs and 24 cytokines had enough cis‐eQTLs to be included in the MR analyses.

### 2.4 Statistical analyses

The two sets of instrumental variables (cis‐pQTLs and cis‐eQTLs) were used separately for evaluating the causal effects of circulating cytokines on RCC in both genders. Wald ratios were calculated to estimate the causal associations of different SNPs with the outcomes. When more than three SNPs were available for a cytokine, the random‐effect inverse‐variance weighted (IVW) MR method was used to pool the estimates from different SNPs, otherwise, a random‐effect IVW was used. Several other MR analysis methods were further performed as sensitivity analyses. MR‐Egger is a method that can detect and correct potential horizontal pleiotropy.^21^ The weighted median is a method that can give causal estimates when up to 50% of instrumental variables are invalid.^22^ MR‐PRESSO method can identify outliers from the MR analyses and gives estimates after excluding the outliers, thus reducing the risk of heterogeneity and horizontal pleiotropy.^23^ The heterogeneity of the MR analyses was quantified with Cochrane's Q value. Horizontal pleiotropy is when a genetic variant affects the outcome by influencing other factors independent of the exposure of interest. In this study, the horizontal pleiotropy is assessed with the intercept test of the MR‐Egger analyses.^21^

All statistical analyses were two‐sided. A *p* value <0.002 (0.05/30, Bonferroni adjusted for 30 cytokines) was considered statistically significant, and a *p* value between 0.05 and 0.002 was considered suggestively significant. All analyses were performed on the R platform (4.1.0) by using *Two‐Sample MR*,^24^ *Mendelian Randomization*,^25^ and *MR‐PRESSO* ^23^ packages.

## 3 RESULTS

### 3.1 Circulating cytokine levels and RCC

**FIGURE 2:**
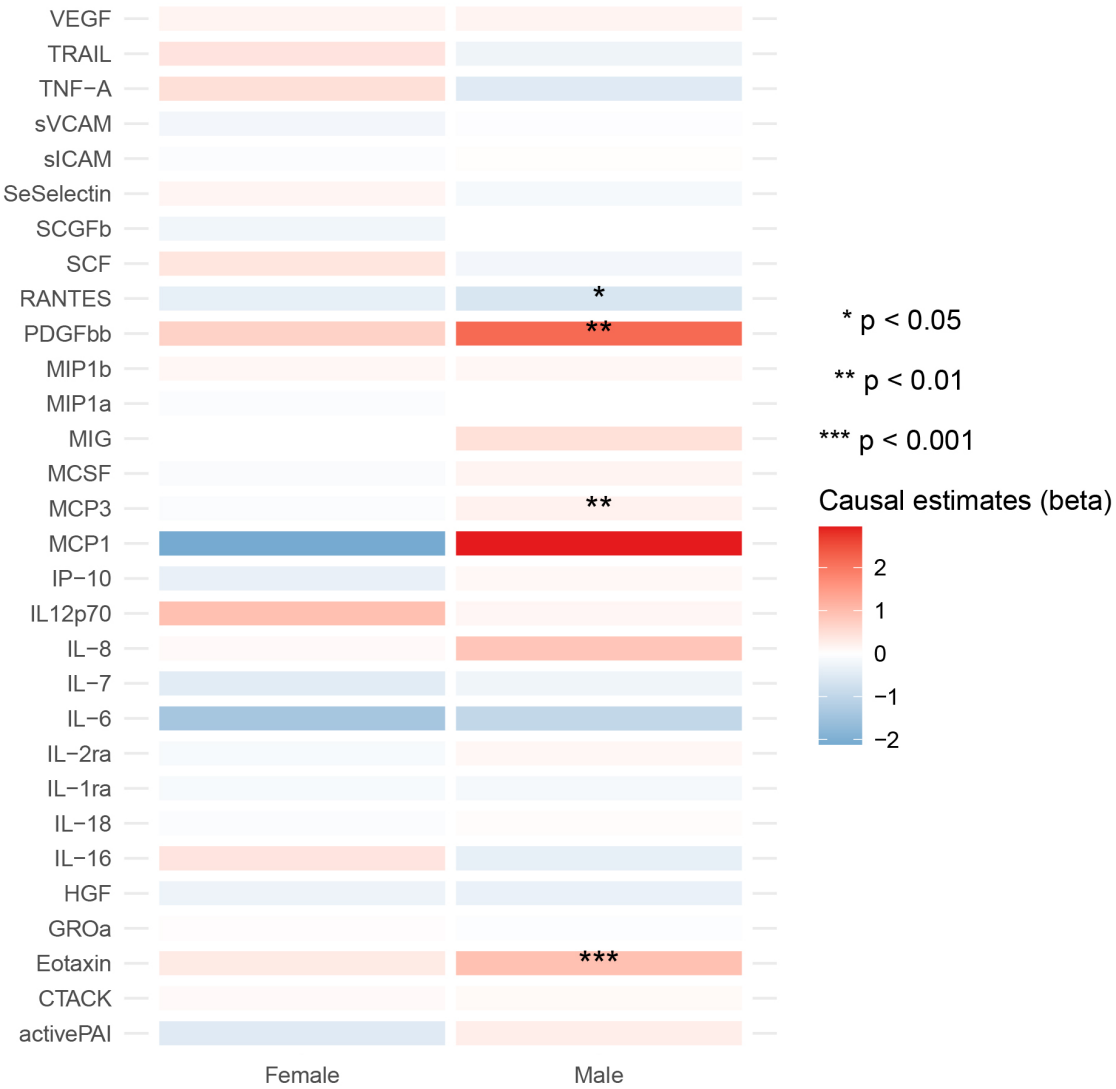
Heatmap showing the causal effects of circulating cytokine levels proxied with cis‐pQTLs on the risk of RCC in males and females by using the IVW method. Cis‐pQTL, cis protein quantitative trait loci; RCC, renal cell carcinoma; IVW, inverse‐variance weighted.

In total 30 cytokines had enough cis‐pQTLs, to be included in the MR analyses. Among the 30 cytokines, only Eotaxin was found to have a significant causal effect on the risk of RCC in males (Odds ratio [OR] = 2.546, 95% confidence interval [CI] = 1.617–4.010, *p* value = 5.496 × 10^−5^) with IVW method (Figure 2, Tables S1–S4). However, this effect did not remain significant in the females (OR = 1.352, 95% CI = 0.766–2.388, *p* value = 0.298) (Figure 2, Tables S5–S8). Three other cytokines presented suggestively significant causal associations with RCC in males, including MCP3 (Monocyte chemotactic protein‐3, OR = 1.211, 95% CI = 1.065–1.377, *p* value = 0.003 with IVW), PDGFbb (Platelet‐derived growth factor bb, OR = 8.720, 95% CI = 1.683–45.172, *p* value = 0.010 with Wald ratio), and RANTES (Regulated on activation, normal T cell expressed and secreted, OR = 0.535, 95% CI = 0.321–0.892, *p* value = 0.016 with Wald ratio) (Figure 2, Tables S1, S5). Similarly, none of these associations remained in the analyses with females (Figure 2, Table S5). No horizontal pleiotropy was identified in these analyses with MR‐Egger regression (Tables S2, S6). The heterogeneity of MR analyses and the used SNPs are shown in Tables S3, S4, S7, and S8.

### 3.2 Cytokines' gene expression and RCC

**FIGURE 3:**
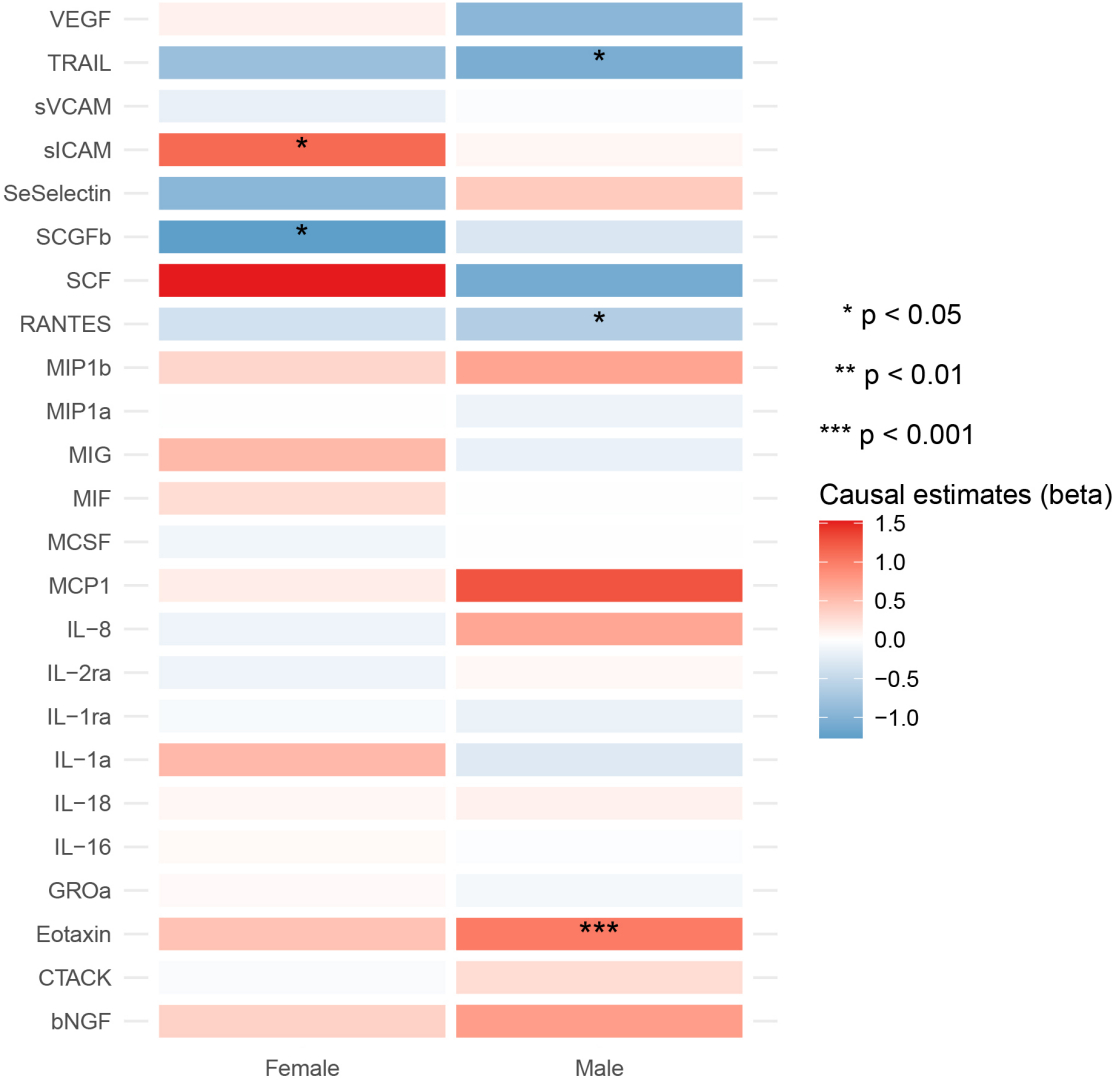
Heatmap showing the causal effects of circulating cytokine gene expressions proxied with cis‐eQTLs on the risk of RCC in males and females by using the IVW method. Cis‐eQTL, cis expression quantitative trait loci; RCC, renal cell carcinoma; IVW, inverse‐variance weighted.

Twenty‐four cytokines had enough cis‐eQTLs to be included in the MR analyses. Similarly, the gene expression of Eotaxin was found significantly associated with the risk of RCC in males (OR = 2.730, 95% CI = 1.554–4.797, *p* value = 4.770 × 10^−4^), but not in females by using the IVW method (Figure 3, Tables S9–S16). Besides, the expression of TRAIL (TNF‐related apoptosis‐inducing ligand) and RANTES were shown to have a suggestive effect on RCC in males, while the expression of sICAM (Soluble intercellular adhesion molecule) and SCGFb (Stem cell growth factor‐β) were found to have a suggestive effect on the risk of RCC in females (Figure 3, Tables S9, S13). Horizontal pleiotropy, heterogeneity and used SNPs in the MR analyses are presented in Tables S10–S12, S14–S16.

### 3.3 Eotaxin and RCC

**FIGURE 4:**
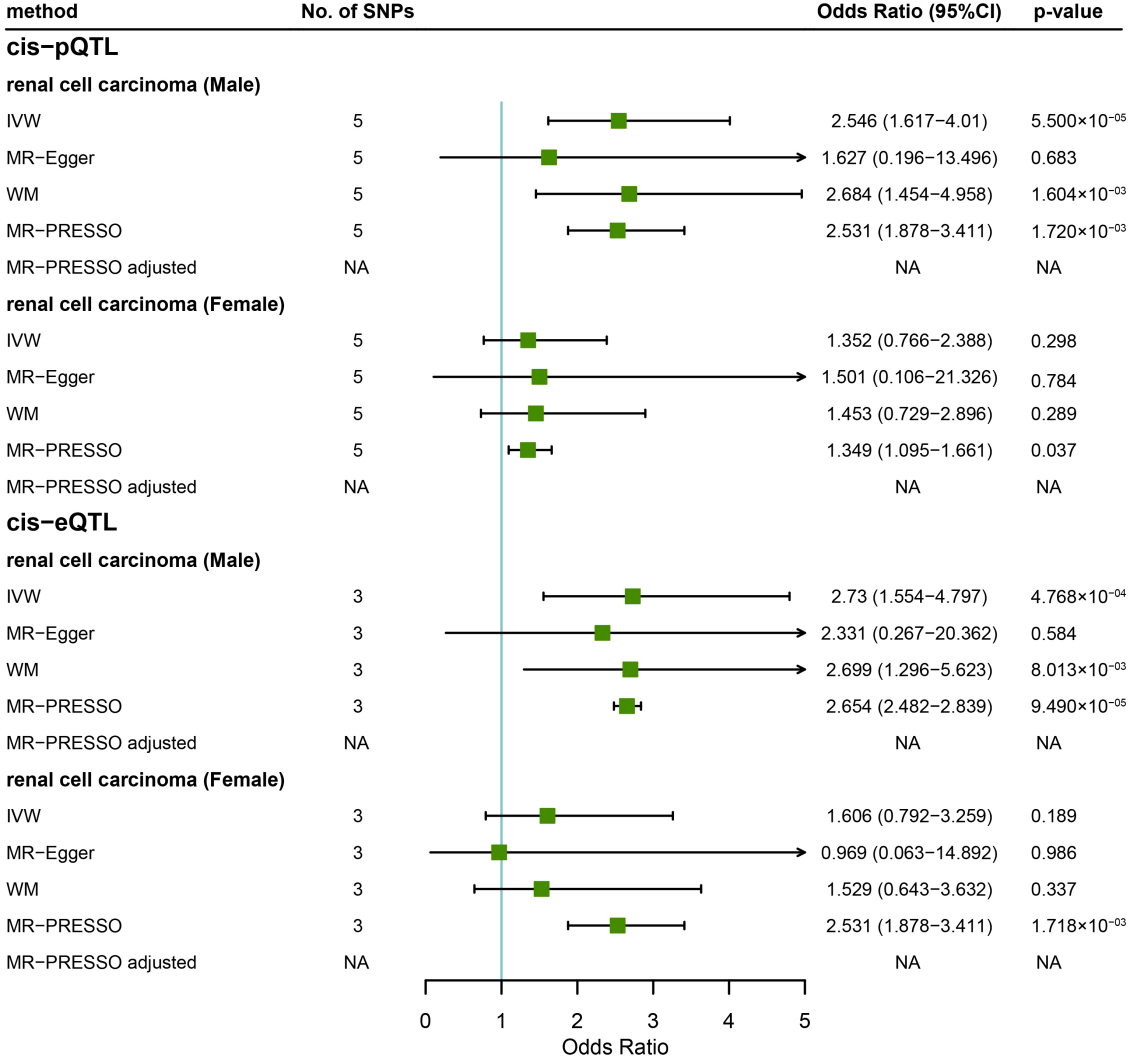
Forest plot showing the causal effects of Eotaxin protein level and gene expression on the risk of RCC in males and females by using IVW, MR‐Egger, WM, or MR‐PRESSO methods. SNP, single nucleotide polymorphism; IVW, inverse‐variance weighted; MR, Mendelian randomization; WM, weighted median.

To confirm the causal associations that we observed between cytokines and RCC, we further performed several sensitivity analysis methods. The level of Eotaxin proxied by cis‐pQTLs remained consistent by using weighted median (OR = 2.684, 95% CI = 1.454–4.958, *p* = 1.604 × 10^−3^) and MR‐PRESSO (OR = 2.531, 95% CI = 1.878–3.411, *p* = 1.720 × 10^−3^) methods (Figure 4). No outlier was identified with the MR‐PRESSO method. Eotaxin showed a tendency to increase the risk of RCC in males with the MR‐Egger method but with a wider confidence interval (Figure 4). Similarly, Eotaxin gene expression proxied by cis‐eQTL was significantly associated with RCC in males with weighted median and MR‐PRESSO methods (Figure 4). To rule out the possibility of reverse causality, we further performed a reverse MR to assess the causal effects of genetically proxied RCC on the Eotaxin protein level. No significant causal effects of RCC on Eotaxin protein level were found with the IVW method (male: beta = −0.015, se = 0.030, *p* = 0.616, female: beta = −0.010, beta = 0.027, *p* = 0.721).

## 4 DISCUSSION

In the current study, we have assessed the effect of circulating inflammatory cytokines on the risk of RCC in males and females with a two‐sample MR method. The consistent results in our analyses highlighted Eotaxin as causally associated with increased RCC risk in males, but not in females. As currently there are no specific molecular biomarkers for the early detection and targeted treatment of RCC, our findings may provide a potential target for related studies in the future.

Cytokines have been identified as important players in the development and progression of neoplasms via promoting the migration and proliferation of tumor cells.^10^, ^26^ Cytokine‐based therapy has been proposed and explored as a potential treatment for solid tumors.^10^, ^26^ For this purpose, it appears critical to identify cytokines that have a causal association with tumors. MR was previously used to investigate the causal effects of cytokines with cancers including prostate cancer, breast cancer, endometrial cancer, lung cancer, and ovarian cancer etc.^14^, ^27^, ^28^ These studies based on an MR design have identified multiple cytokines as associated with different cancer risks.^14^, ^27^, ^28^ However, the effects of cytokines on RCC risk have not been explored yet.

RCC is characterized by high vascularization and an immunological cancer background.^11^, ^29^ Cytokine and chemokine burst is an important pathological factor known to be associated with the rapid proliferation of RCC.^29^ Cytokines play a pivotal role in regulating the tumor microenvironment and antitumor immunity in patients with RCC.^30^ Furthermore, a previous observational study using urine and tumor tissues from RCC patients identified that different cytokines were elevated in the tumor tissues at different stages of RCC. Due to the distinction between RCC incidence, histological subtypes, and prognosis in different genders, we investigated the effects of circulating cytokines on RCC in different genders.^31^ Interestingly, several cytokines were identified as having a significant or suggestively significant causal effect on RCC, while the effects were different in males and females. Both protein levels and gene expression of Eotaxin were found to be positively correlated with the risk of RCC in males but not in females (Figure 4). Several reasons may be contributing to the gender difference. Firstly, this study included RCC patients of all histological subtypes (Table 1), however, females tend to have lower grades and earlier stages of RCC than males, which may have different responses to cytokines.^31^ Secondly, some studies proposed that hormones may play a role in the progression of RCC as well, while sex hormones are also known to be able to modify cytokine responses.^31^ Besides, differences in lifestyle habits such as smoking may also contribute to this. After all, more studies are needed to better explain the exact mechanisms. As the primary ligand of CCR3 (C‐C Motif Chemokine Receptor 3), Eotaxin‐1 has been identified as involved in the tumor‐associated inflammation that facilitates the development and progression of CCR3‐positive RCC.^2^ Interestingly, the level of CCR3 was found to be correlated with the grade of malignancy of RCC.^30^ Besides, the existence of the CCR3/Eotaxin‐1 loop in T‐cell lymphomas has been reported to induce the growth of malignant cells.^32^ Observational studies have reported a higher level of Eotaxin in the urine of RCC patients, and 3 days after nephrectomy, the levels dropped immediately.^11^ Apart from RCC, Eotaxin was also reported to be associated with tumorigenesis in several other malignancies including colon cancer, oral squamous cell carcinoma, and prostate cancer.^33^, ^34^, ^35^ Consistency in the evidence further validated the robustness of our findings.

**TABLE 1:** Detailed information on data sources of the included GWAS studies.

Traits	Consortium	Cases	Control	Sample size	Year	Population	Pubmed ID	Definition
Circulating cytokines	NA	NA	NA	Up to 31,112	2022	European	35012533	NA
Renal cell carcinoma	IARC	3227 males and 1992 females	4916 males and 3095 females	13,230	2019	European	31231134	Invasive RCC (International Classification of Disease for Oncology second and third Edition topography code C64), including all histological subtypes, diagnosed in adults (≥aged 18 years)

Abbreviations: GWAS, genome‐wide association studies; RCC, renal cell carcinoma.

There are several advantages of our study. Firstly, this is to our knowledge the first study investigating the causal effects of circulating cytokines on the risk of RCC with MR methods. Our analyses estimated the associations between circulating cytokines with RCC while minimizing the bias from reverse causality and confounding factors. Besides, our estimations were obtained from both males and females separately. Considering the different incidence rates, histological subtypes, and prognosis of RCC between males and females, our results may provide evidence of the different etiology of RCC in the two genders. Furthermore, unbalanced horizontal pleiotropy is a common bias that may distort causal estimates from the MR analyses. In our analyses, the instrumental variables employed in the analyses were cis SNPs, which minimized the risk of bias from horizontal pleiotropy. We evaluated the potential horizontal pleiotropy with the MR‐Egger intercept test and MR‐PRESSO outlier identification as well. Lastly, we have also used several sensitivity tests to validate the robustness of our results and restricted the study population to European ancestry to reduce the bias from population stratification.

However, there still exist several limitations in our study to be considered when interpreting our results. First of all, restricting the study to the European population also restricted the generalization of our conclusions to other populations. Secondly, previous studies have suggested that circulating cytokine levels poorly mimic the tumor cytokine microenvironment. The distinct cytokine profile between plasma and RCC tumor may lead to different conclusions when the analyzed samples were obtained from different tissues. Further studies are needed to explore the relationships between circulating cytokines and tumor cytokine microenvironment, and also the exact mechanistic role of circulating Eotaxin in the development of RCC. Besides, the causal effects of Eotaxin on RCC may still be biased by potential reverse causality. To minimize the risk, we have performed a reverse MR to estimate the causal effects of RCC on Eotaxin levels and no significant causal associations were observed. Lastly, significant heterogeneity was identified in MR analyses of some cytokines on RCC risk, and a random‐effect IVW method was employed to minimize the risk of bias. We also used the MR‐PRESSO method to detect and exclude potential outliers in the analyses.

In conclusion, our study highlighted the genetically proxied higher level of circulating Eotaxin as a causal factor that increased the risk of RCC in males but not in females. Further studies are needed to investigate the exact mechanisms and validate its potential to be used as a drug target for the prevention of RCC.

## AUTHOR CONTRIBUTIONS

**Shuixiang Tao:** Conceptualization (equal); data curation (lead); formal analysis (lead); methodology (lead); supervision (supporting); writing – original draft (lead); writing – review and editing (supporting). **Yiwei Lin:** Data curation (equal); formal analysis (equal); writing – original draft (supporting); writing – review and editing (supporting). **Shengqiang Huang:** Data curation (supporting); formal analysis (supporting); writing – review and editing (supporting). **Shen Lin:** Data curation (supporting); formal analysis (supporting); writing – review and editing (supporting). **Ke Jin:** Data curation (supporting); formal analysis (supporting); writing – review and editing (supporting). **Hong Chen:** Conceptualization (lead); supervision (lead); writing – original draft (supporting); writing – review and editing (lead).

## FUNDING INFORMATION

This study was supported by grants from the Zhejiang Medical and Health Science and Technology Project (2023XY070).

## CONFLICT OF INTEREST STATEMENT

The authors have no relevant financial or nonfinancial interests to disclose.

## ETHICS STATEMENT

This study used only published summary‐level data from studies involving human participants, with written informed consent and approved by their respective local ethics review committees.

## Supporting information


Tables S1–S16

## Data Availability

All data generated or analyzed during this study are included in this published article and its supplementary information files.
